# Protective function of interleukin‐22 in pulmonary fibrosis

**DOI:** 10.1002/ctm2.509

**Published:** 2021-08-26

**Authors:** Peiyu Gu, Dandan Wang, Ji Zhang, Xin Wang, Zhiyong Chen, Lina Gu, Mengying Liu, Fanqing Meng, Jun Yang, Hourong Cai, Yonglong Xiao, Yin Chen, Mengshu Cao

**Affiliations:** ^1^ Department of Respiratory and Critical Care Medicine Nanjing Drum Tower Hospital, The Affiliated Hospital of Nanjing University Medical School Nanjing Jiangsu China; ^2^ Department of Respiratory and Critical Care Medicine Nanjing Drum Tower Hospital Clinical College of Nanjing Medical University Nanjing Jiangsu China; ^3^ Department of Rheumatology and Immunology, Nanjing Drum Tower Hospital The Affiliated Hospital of Nanjing University Medical School Nanjing Jiangsu China; ^4^ Wuxi Transplant Center Wuxi People's Hospital Affiliated to Nanjing Medical University Wuxi Jiangsu China; ^5^ Department of Respiratory and Critical Care Medicine, Nanjing Drum Tower Hospital Clinical College of Traditional Chinese and Western Medicine Nanjing University of Chinese Medicine Nanjing China; ^6^ Department of Pathology, Nanjing Drum Tower Hospital The Affiliated Hospital of Nanjing University Medical School Nanjing Jiangsu China; ^7^ Department of Pharmacology and Toxicology School of Pharmacy; University of Arizona Tucson AZ; ^8^ Asthma & Airway Disease Research Center University of Arizona Tucson AZ

**Keywords:** fibrosis, IL‐22, IPF, TGF‐β

## Abstract

Idiopathic pulmonary fibrosis (IPF) is a chronic and progressive scarring disease with unknown etiology. The evidence of a pathogenic role for transforming growth factor‐beta (TGF‐β) in the development and progression of IPF is overwhelming. In the present study, we investigated the role of interleukin‐22 (IL‐22) in the pathogenesis of IPF by regulating the TGF‐β pathway. We measured parameters and tissue samples from a clinical cohort of IPF. IL‐22R knock out (IL‐22RA1^−/−^) and IL‐22 supplementation mouse models were used to determine if IL‐22 is protective in vivo. For the mechanistic study, we tested A549, primary mouse type II alveolar epithelial cell, human embryonic lung fibroblast, and primary fibroblast for their responses to IL‐22 and/or TGF‐β1. In a clinical cohort, the expression level of IL‐22 in the peripheral blood and lung tissues of IPF patients was lower than healthy controls, and the lower IL‐22 expression was associated with poorer pulmonary function. IL‐22R^−/−^ mice demonstrated exacerbated inflammation and fibrosis. Reciprocally, IL‐22 augmentation by intranasal instillation of recombinant IL‐22 repressed inflammation and fibrotic phenotype. In vitro, IL‐22 treatment repressed TGF‐β1 induced gene markers representing epithelial‐mesenchymal‐transition and fibroblast‐myofibroblast‐transition, likely via the inhibition of TGF‐β receptor expression and subsequent Smad2/3 activation. IL‐22 appears to be protective against pulmonary fibrosis by inhibiting TGF‐β1 signaling, and IL‐22 augmentation may be a promising approach to treat IPF.

## Graphical abstract

We measured parameters and tissue samples from a clinical cohort of IPF?IL‐22R knock out (IL‐22R^−/−^) and IL‐22 supplementation mouse models?and A549, human embryonic lung fibroblast and primary fibroblast. We suggested that IL‐22 appears to be protective against pulmonary fibrosis by inhibiting TGF‐?1 signaling, and IL‐22 augmentation may be a promising approach to treat IPF.

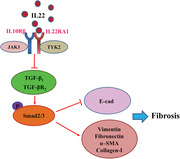

## 1 INTRODUCTION

In general, pulmonary fibrosis represents a chronic disease with the development of irreversible scarring, lung remodeling, and graduate loss of pulmonary function. The triggers for causing injury and initiating/maintaining the fibrotic process are highly controversial or completely unknown.^1^ Idiopathic pulmonary fibrosis (IPF) has an unknown etiology and poor prognosis with a median survival of 2–3 years after diagnosis.^2^ Despite intensive drug development effects, there is no preventative or therapeutic modality that can stop the disease progression of IPF. Two drugs currently in clinical use: nintedanib^3^ and pirfenidone^4^ can only slow, but not stop, the decline of pulmonary function. Of note, both drugs do not completely inhibit the TGF‐β pathway, an important determinant in IPF pathogenesis.^5^ In the end, lung transplantation is likely the only option for advanced IPF patients, but it causes significant complications and has a disappointing median survival rate of 5.8 years that is far behind other solid organ transplantations.^6^ Thus, a new therapeutic intervention for IPF is urgently needed.

Transforming growth factor‐β1 (TGF‐β1) is considered a crucial mediator in pulmonary fibrosis by activating its downstream small mother against decapentaplegic (Smad) signaling. The primary effector cells in IPF are myofibroblasts, which produce a high amount of collagen and are characterized by the presence of α‐smooth muscle actin (α‐SMA) stress fibers. These cells may be derived by activation/proliferation of resident lung fibroblasts, epithelial‐mesenchymal transition, or recruitment of circulating fibroblastic stem cells (fibrocytes).^7^ Abundant literature supports a critical role of the TGF‐β signaling pathway in the development and progression of IPF. TGF‐β can be produced by alveolar epithelial cells, fibroblasts, inflammatory cells, and macrophages in the lung, drive the epithelial‐mesenchymal transition and fibroblast‐to‐MF differentiation and induce extracellular matrix production.^7^ TGF‐β1 directly activates Smad signaling thereby inducing pro‐fibrotic gene expression. TGF‐β pathway blockade is a powerful therapeutic modality, in particular, to reduce myofibroblast activity and consequently pulmonary fibrogenesis.^7^ Thus, inhibiting TGF‐β signaling is likely a promising therapy for treating IPF.

IL‐22 is a member of the IL‐10 family and is produced by cells of the innate and adaptive immune system including innate lymphoid cells, natural killer cells, activated Th1, Th17, Th22, γδ T cells^8^ ^,^ and macrophages.^9^ Its receptor, a heterodimer consisting of IL‐22R1 and IL‐10R2, is universally expressed on both hematopoietic and non‐hematopoietic cells. IL‐22 is implicated in many respiratory diseases, including COPD^10^ and fibrosis.^11^ However, the function of IL‐22 can be both pro‐ or anti‐fibrosis, and these opposite functions are context‐, species‐ and disease‐dependent.^11^, ^12^ For example, the protective role of γδ T‐cell‐derived IL‐22 in pulmonary fibrosis has first been shown in a hypersensitive pneumonitis model induced by repeated exposure to *Bacillus subtilis*.^13^ In that study, IL‐22 inhibition enhanced collagen deposition while treatment with recombinant IL‐22 inhibited lung fibrosis, suggesting that IL‐22 might be protective. This notion has been further supported by evidence from more relevant bleomycin (BLM) induced lung fibrosis model, in which treatment with anti‐IL‐22 neutralizing antibody exacerbated airway inflammation and enhanced expressions of a number of fibrotic biomarkers.^14^ Contrary to these findings, an earlier study showed that the lack of IL‐22 in the knockout (KO) model or anti‐IL‐22 antibody treatment in wild‐type (WT) mice actually ameliorated BLM‐induced disease.^15^ Thus, IL‐22 function in the mouse model of pulmonary fibrosis has not been completely defined. Furthermore, clinical evidence linking IL‐22 and pulmonary fibrosis is still scarce.

In this study, we have extended the current literature by examining the clinical association between IL‐22 and IPF. We further defined the role of IL‐22 using a combinatorial approach including IL‐22 receptor KO (IL‐22R ^−/−^), IL‐22 augmentation by intranasal instillation of recombinant IL‐22 and in vitro cell culture. By this rigorous study design, we have for the first time demonstrated the inverse relationship between IL‐22 expression and disease severity of IPF in the clinical cohort, and established the causal role of IL‐22 in the protection against pulmonary fibrosis by inhibiting the TGFβ pathway.

## 2 MATERIALS AND METHODS

### 2.1 Study population

Peripheral blood samples were obtained from 24 patients of IPF (21 male and 3 female, average age 67.2±9.8 years) and 16 healthy volunteers (12 male and 4 female, average age 58.6 ± 13.8 years). There was no significant difference in gender or age between the two groups (*p* = 0.407 and 0.136, respectively). 54.17% of IPF patients (13/24) had a smoking history. The common comorbidities of these patients were a respiratory failure (11/24, 45.83%), diabetes (6/24, 25%), hypertension (5/24, 20.83%), Coronary heart disease (2/24, 8.33%), liver cirrhosis (2/24, 8.33%) and malignancy (2/24, 8.33%) as shown in Supplemental Table 1. Eight lung specimens were collected through open lung biopsy (OLB) (*n* = 4, patients with lung cancer, normal lung tissues adjacent to cancer) or lung transplantation (*n* = 4, IPF patients) from Nanjing Drum Tower hospital and Wuxi People's Hospital, Nanjing Medical University during the same time. The diagnosis of IPF was according to the criteria of the American Thoracic Society and European Respiratory Society in 2011.^16^ Clinical data was obtained from medical records on admission. Chest high resolution computed tomography (HRCT) was performed with 1.0–1.5 mm thick sections and appropriate window settings (window width: 1600, window level: –600). The images were assessed for the presence and extent of ground‐glass opacity, consolidation, traction bronchiectasis, reticulation, honeycombing, and emphysema. The overall extent of abnormalities was determined per lung using a 4‐point scale (0 = no involvement, 1 = 1–25% involvement, 2 = 26–50% involvement, 3 = 51–75% involvement, and 4 = 76–100% involvement) according to the published study.^17^ Those with an inconsistent UIP pattern were excluded from this study. The present study was approved by the Ethics Committee of Nanjing Drum Tower Hospital in accordance with the Declaration of Helsinki (1989; NO.2016‐160‐01).

### 2.2 Animals

IL‐22R^−/−^ mice in the C57BL/6 background were purchased from Biocenter Oulu (University of Oulu, Finland). Male WT or IL‐22R^−/−^ mice (6–8 weeks) were intratracheally injected with 50 μl normal saline (NS) or BLM (5 mg/kg) (Nippon Kayaku, CO., LTD, Tokyo, Japan) to induced fibrosis. Intranasal instillation of 50 μl recombinant IL‐22 (100 ng)^18^ (582‐ML; R&D Systems, USA) at day 1, day 7, day 14 after the treatment of NS or BLM was used to test the protective function of IL‐22. Intranasal instillation of the reagents, BLM, NS, and IL‐22 was administrated to the animals by endotracheal quantitative reagent delivery device (HRH‐MAG4; Yuyan Instruments, Shanghai, China). All protocols were approved by the Institutional Animal Care and Use Committee (IACUC) of Nanjing Drum Tower Hospital.

**HIGHLIGHTS**

1. Low levels of IL‐22 in IPF patients were associated with worse pulmonary function.
2. IL‐22 protected against bleomycin induced inflammation and lung fibrosis.
3. IL‐22 appeared to elicit its protective function by inhibiting TGF‐β1 signaling.

### 2.3 Histopathological analysis

Histopathological analysis was performed on formalin‐fixed lung biopsy tissues from patients with IPF and mice by hematoxylin and eosin (H&E) and Masson's trichrome staining. Primary antibodies were TGF‐β1 (ab92486) and TGF‐βR2 (ab61213; Abcam, USA). The pathological scores of alveolitis at 7 days and fibrosis at 21 days in BLM‐induced mice treatment were calculated based on the method as described previously.^19^ The alveolitis and fibrosis scores were performed blindly by two senior pathologists independently.

### 2.4 Enzyme‐linked immunosorbent assay

The plasma levels of IL‐22 (D2200; R&D Systems, USA) were measured by enzyme‐linked immunosorbent assay according to the manufacturer's protocols.

### 2.5 Cell culture

In vitro, we tested four different cells, type II alveolar epithelial cell (AT2) like cell line (A549; Shanghai Cell Bank, China), primary mouse AT2 cells (CP‐M003; Wuhan Procell, China), human embryonic lung fibroblast (HELF; Shanghai Cell Bank, China) and primary fibroblasts (FB) from lung cancer patients (normal lung tissues adjacent to cancer). The isolation and culture of primary FB followed the protocol in the previous report.^20^ Cells were serum‐starved for 24 h, then stimulated with recombinant human IL‐22 (10 ng/ml)^21^ (782IL; R&D Systems, USA) and/or recombinant human TGF‐β1 (5 ng/ml) (240­B; R&D Systems, USA) with the concentrations indicated in each experiment for 72 or 2 h.

### 2.6 Quantitative real‐time PCR

Total RNA was isolated from frozen cells using TRIZOL Reagent (15596026 and 15596018; Invitrogen, USA). Real‐time PCR was performed as described previously.^22^ The primes (Table 1) were designed and synthesized by TAKALA Biotechnology Co, Ltd (Dalian, China).

**TABLE 1:** Real‐time PCR primers

**Gene**	**Forward (5′to 3′)**	**Reverse (5′** to **3′)**
Human IL‐22	TTCCAGCAGCCCTATATCACC	GCTCACTCATACTGACTCCGTG
Human TGF‐β1	TCCTGGCGATACCTCAGCAA	GCTAAGGCGAAAGCCCTCAA
Human TGF‐βR2	GAAATTCCCAGCTTCTGGCTCA	CTGTCCAGATGCTCCAGCTCAC
Human GAPDH	GCACCGTCAAGGCTGAGAAC	TGGTGAAGACGCCAGTGGA
Human α‐SMA	GGCTTCTCTATCTACCTTCC	ACATTCACAGTTGTGTGCTA
Human E‐cadherin	AGTGACGAATGTGGTACCTTTTGA	TGAAGGGAGATGTTTGGGGAGGAAGGTC
Human vimentin	TCTGGATTCACTCCCTCTGGTT	ATCGTGATGCTGAGAAGTTTCGT
Human collagen‐I	GAGGGCCAAGACGAAGACATC	CAGATCACGTCATCGCACAAC
Mouse TGF‐β1	TACGGCAGTGGCTGAACCAA	CGGTTCATGTCATGGATGGTG
Mouse TGF‐βR2	TTAACATGATGTCATGGCCAGCG	AGACTTCATGCGGCTTCTCACAGA
Mouse GAPDH	AAATGGTGAAGGTCGGTGTGAAC	CAACAATCTCCACTTTGCCACTG

Abbreviations: IL, interleukin; SMA, smooth muscle actin; TGF, transforming growth factor.

### 2.7 Western blot

Western blot analysis was performed as described previously.^22^ Primary antibodies were anti‐IL‐22 (ab181007; Abcam), anti‐TGF‐β1 (ab92486; Abcam), anti‐α‐SMA (ab7818; Abcam), E‐cad (3195S; CST, USA), TGF‐βR2 (ab61213; Abcam), Vimentin (5741S), Smad2/3 (8685S) and P‐smad2/3 (8828S; CST), GADPH (14C10; CST), Vimentin (ab92547; Abcam), Fibronectin (FN) (610077; BD Systems, USA), Collagen‐I (ab34717; Abcam). Secondary antibodies were goat anti‐rabbit and rabbit anti‐mouse secondary antibodies (7076S and 7074S; CST).

### 2.8 Statistical analysis

Data are presented as mean ± SD for continuous variables or percentages for categorical variables. Chi‐square and Fisher's exact tests were used for categorical data, and the unpaired *t*‐test and Kruskal–Wallis test were used for continuous data. For multiple comparisons, one‐way ANOVA was used. *p* < 0.05 was accepted as statistically significant. Mann‐Whitney *U*‐test was employed for data of non‐Gaussian distribution. All experiments were repeated at least three times. GraphPad Prism software (GraphPad Software Inc., San Diego, CA, USA) was used for mapping the figures.

## 3 RESULTS

### 3.1 The expression of IL‐22 was lower in the peripheral blood of IPF patients and correlated negatively with the disease severity

**FIGURE 1:**
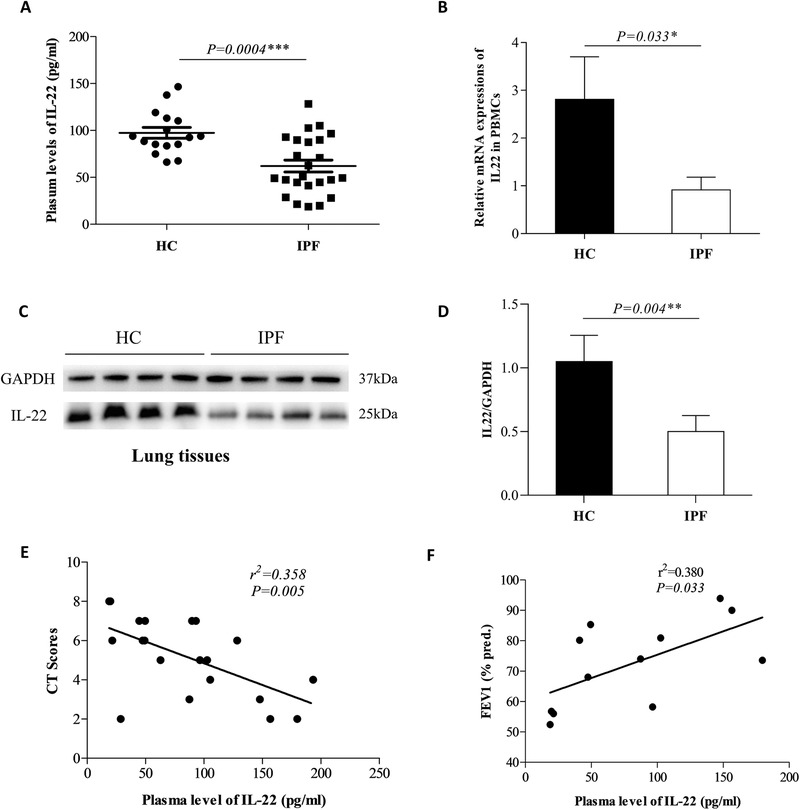
Lower expression levels of interleukin‐22 (IL‐22) in idiopathic pulmonary fibrosis (IPF) were associated with increased disease severity. (A) The plasma IL‐22 level in healthy controls (*n* = 16) and IPF patients (*n* = 24). (B) The mRNA expressions of IL‐22 in peripheral blood mononuclear cells (PBMC) of healthy controls (HC, *n* = 16) and IPF patients (*n* = 24) (The values were normalized to GAPDH). (C) Western blot of analysis of protein expression of IL‐22 in the lung tissues. (D) The quantification of (C). (E) The plasma IL‐22 level was negative with computed tomography (CT) scores in IPF patients (*n* = 20). (F) The plasma IL‐22 level was positive with forced expiratory volume at 1 s predicted percent (FEV1% pred) in IPF patients

IL‐22 level was found to be significantly lower in plasma samples from IPF patients (*n* = 24) as compared with healthy controls (HC, *n* = 16) (58.19 ± 7.20 pg/ml versus 97.47 ± 5.74 pg/ml, *p *< 0.001; Figure 1A). Consistently, IL‐22 mRNA expression in PBMCs of IPF patients was also lower than normal controls (*p *< 0.05; Figure 1B). The results of WB showed that the expressions of IL‐22 in the lung tissues from IPF patients were significantly decreased compared with the healthy controls (HC; Figure 1C, D). Correlation analysis showed that plasma IL‐22 levels were negatively correlated with CT scores of IPF (*r* ^2 ^= 0.358, *p *= 0.005; Figure 1E), and positively correlated with a pulmonary function parameter, FEV1% pred (*r* ^2 ^= 0.380, *p *= 0.033; Figure 1F), suggesting a negative correlation between blood IL‐22 and the disease severity of IPF.

### 3.2 Increased activity of the TGF‐β pathway was observed in the lung of IPF patients

**FIGURE 2:**
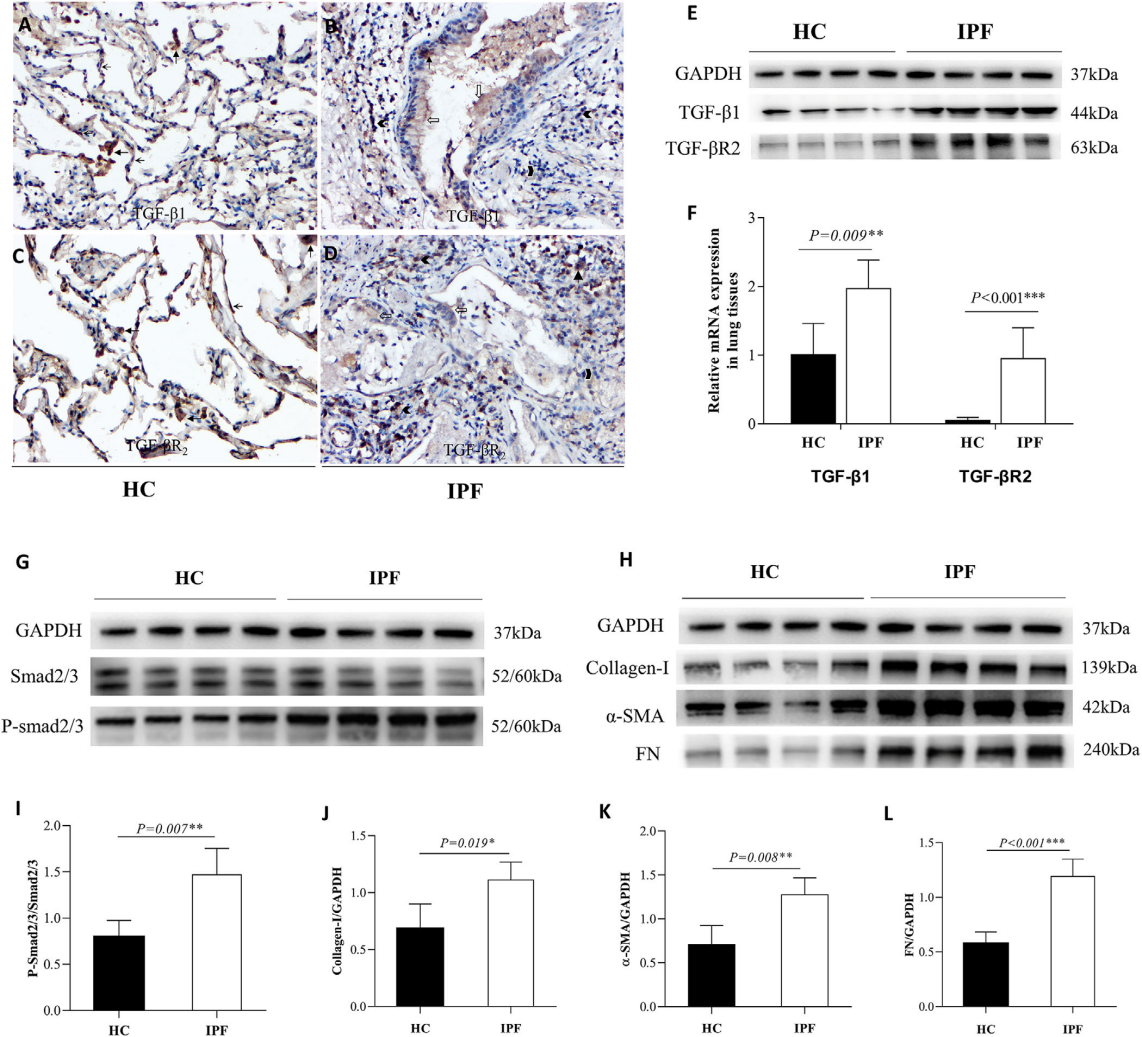
Increased expressions of transforming growth factor (TGF)‐β_1_, TGF‐βR_2_, P‐smad2/3, and Collagen‐I in the lung of patients with idiopathic pulmonary fibrosis (IPF). (A, B) The expressions of TGF‐β_1_ in healthy controls (HC) and IPF patients by immunohistochemistry (IHC, ×200). (C, D) The expressions of TGF‐βR_2_ in HC and IPF patients by IHC (×200). (E) Western blot analysis in the lung tissues of HC (*n* = 4) and IPF patients (*n* = 4). (F) mRNA expression analysis of TGF‐β_1_ and TGF‐βR_2_ in HC and IPF patients by real‐time PCR in lung tissues. (G–H) Western blot analysis of protein levels of P‐smad2/3, Collagen‐I, α‐smooth muscle actin  (α‐SMA), and fibronectin (FN) in lung biopsy tissues from HC and IPF patients (HC, *n* = 4 and IPF patients, *n* = 4). (I–L) The quantification of (G–H). (Notes: phagocytes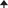, type II AEC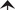, bronchial mucosa epithelial cells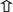, inflammatory cell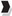)

As the TGF‐β pathway has been demonstrated to play important role in IPF pathogenesis, we tested its signaling components in lung tissue samples from IPF. Using Immunohistochemistry and WB, TGF‐β1 (Figure 2B, E) and a major TGF‐β receptor‐2 (TGF‐βR2) (Figure 2D, E) were increased in IPF lung tissues as compared with HC (Figure 2A, C). Consistently, mRNA levels of TGF‐β1 and TGF‐βR2 were also elevated in lung tissues of IPF patients (Figure 2F). Supporting this notion, the key downstream transcriptional factor Smad2/3 was highly activated in IPF as demonstrated by increased P‐smad2/3 (Figure 2G, I), and there was also a marked increase of Collagen‐I, α‐SMA, and FN levels in IPF lung (Figure 2H, J–L). Thus, the activity of the TGF‐β pathway in the IPF lung appeared to be significantly higher than in the normal lung.

### 3.3 The lack of IL‐22 signaling increased pathology in BLM induced fibrosis model

**FIGURE 3:**
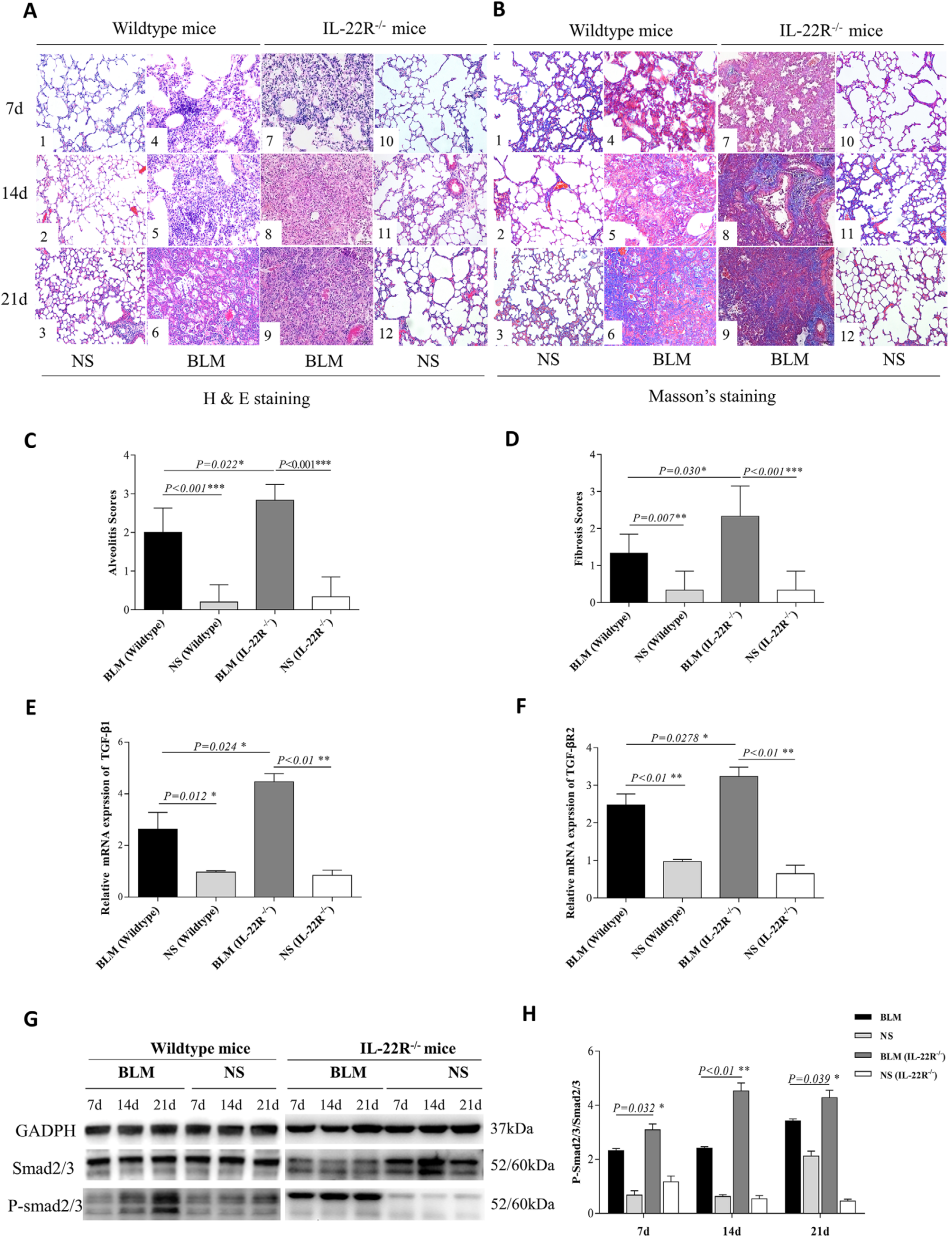
Elevated bleomycin (BLM)‐induced inflammation and fibrosis in interleukin (IL)‐22R^−/−^ mice. Mice were intratracheally injected with 50 μl normal saline (NS) or BLM (5 mg/kg) to induce fibrosis at day 0. Each set of experiments was repeated three times. Six mice per group were used. (A1–6) The pathological changes of lung tissues on days 7, 14, and 21 in NS and BLM treated wild‐type (WT) mice by HE staining (×200). (A7–12) The pathological changes of lung tissues on days 7, 14, and 21 in BLM and NS treated IL‐22R**^−/^** ^−^ mice by HE staining (×200). (B1–6) The pathological changes of lung tissues at day 7, 14, and 21 days in NS and BLM treated WT mice by Masson's trichrome (MS) staining (×200). (B7–12) The pathological changes of lung tissues on days 7, 14, and 21 in BLM and NS treated IL‐22R**^−/‐^** mice by MS staining (×200). (C) Comparison of the pathological scores of alveolitis at 7 days after treatment with BLM in WT mice and IL22R**^−/^** ^−^ mice. (D) Comparison of the pathological scores of fibrosis at 21 days after treatment with BLM in WT (*n* = 6) and IL22R**^−/^** ^−^ mice (*n* = 6). (E–F) The mRNA expressions of transforming growth factor (TGF)‐β_1_ and TGF‐βR_2_ in the lungs of BLM, NS treated WT and IL22R**^−/‐^** mice at 21 days. (G–H) The protein expressions of P‐smad2/3 in the lung tissues of BLM, NS treated WT, and IL22R**^−/‐^** mice by western blot analysis

To determine in vivo function of IL‐22, we utilized BLM induced mouse model of pulmonary fibrosis. Using HE staining, severe inflammation in peri‐bronchi and alveolar septum was demonstrated at 7, 14, and 21 days in BLM treated WT mice (Figure 3A 4, 5, 6), but not in NS stimulated WT mice (Figure 3A1–3). Consistently, there was significant collagen deposition in the BLM‐treated group (Figure 3B 4, 5, 6), but not in the control group (Figure 3B 1, 2, 3) as demonstrated by Masson's trichrome (MS) staining. Inflammatory responses and collagen deposition were further exacerbated by BLM treatment in IL‐22R^−/−^ mice (Figure 3A7–9, Figure 3B7–9) compared with WT mice (Figure 3A 4, 5, 6, Figure 3B 4, 5, 6) and NC treatment IL‐22R^−/−^ mice (Figure 3A10–12, Figure 3B10–12). The low magnification of lung tissues in both wildtype and IL‐22R^−/−^ mice are shown in Figure S1A–B. The pathological scores of alveolitis at 7 days (*p* = 0.01, Figure 3C) and fibrosis (*p* = 0.03, Figure 3D) at 21 days after treatment with BLM were increased significantly in IL22R**^−/^** ^−^ mice (*n* = 6) as compared with wild type mice (*n* = 6). At the molecular level, mRNAs of TGF‐β1 and TGF‐βR2 were increased at 21 days in BLM treated WT mice as compared to NS treated WT mice, and their expressions were further enhanced in BLM treated IL‐22R^−/−^ mice (Figure 3E–F). Consistently, the key downstream signaling component, P‐smad2/3 in the TGF‐β pathway was also activated time‐dependently in BLM treated WT mice, and its level was further enhanced in BLM treated IL‐22R^−/‐^ mice (Figure 3G–H). Thus, the lack of IL‐22 signaling appeared to augment the TGF‐β pathway and exacerbate fibrosis in the BLM‐induced fibrosis model.

**FIGURE 4:**
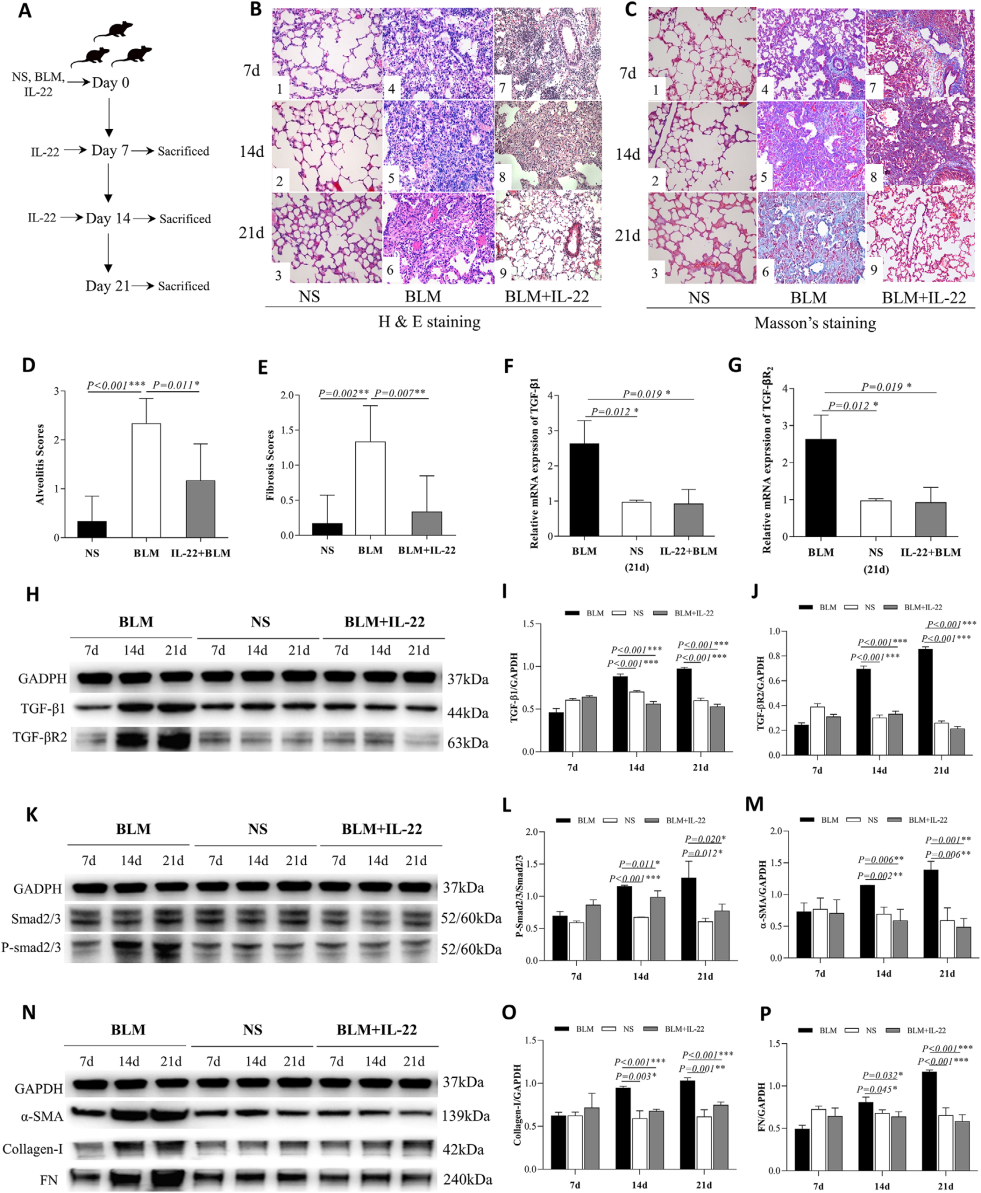
Interleukin (IL)‐22 treatment ameliorated bleomycin (BLM)‐induced inflammation and collagen deposition. (A) Experimental schematic of the animal. Mice were intratracheally injected with 50 μl normal saline (NS) or BLM (5 mg/kg) to induced fibrosis at day 0. Intranasal instillation of 50 μl recombinant IL‐22 (100 ng) was used on day 0, day 7, and day 14 after the treatment with BLM, parts of mice were sacrificed at day 7, day 14, and day 21. Each test was repeated three times. Six mice per group were used. (B1–3) The pathological changes of lung tissues on days 7, 14, and 21 in NS treated mice by HE staining (×200). (B4–6) The pathological changes of lung tissues on days 7, 14, and 21 in BLM treated mice by HE staining (×200). (B7–9) The pathological changes of lung tissues on days 7, 14, and 21 in both BLM and IL‐22 treated mice by HE staining (×200). (C1–3) The pathological changes of lung tissues at day 7, 14, and 21 days in NS treated mice by MS staining (×200). (C4–6) The pathological changes of lung tissues at day 7, 14, and 21 days in BLM treated mice by MS staining (×200). (C7–9) The pathological changes of lung tissues at day 7, 14, and 21 days in both BLM and IL‐22 treated mice by MS staining (×200). (D) Comparison of the pathological scores of alveolitis at 7 days in BLM (*n* = 6) and both BLM and IL‐22 treated mice (*n* = 6). (E) Comparison of the pathological scores of fibrosis at 21 days in BLM (*n* = 6) and both BLM and IL‐22 treated mice (*n* = 6). (F–G) The mRNA expression of transforming growth factor (TGF)‐β_1_ and TGF‐βR_2_ in lung tissues of BLM, NS, and both BLM and IL‐22 treated mice at day 21 by real‐time PCR. (H) The protein expression of TGF‐β_1_ and TGF‐βR_2_ in lung tissues of BLM, NS, both BLM and IL‐22 treated mice by WB. (I–J) The quantification of (G). (K) The protein expression of P‐smad2/3 in lung tissues of BLM, NS, and both BLM and IL‐22 treated mice by WB. (L) The quantification of (K). (N) The protein expression of α‐SMA, Collagen‐I, and fibronectin (FN) in lung tissues of BLM, NS, and both BLM and IL‐22 treated mice by WB. (M, O–P) The quantification of (N)

**FIGURE 5:**
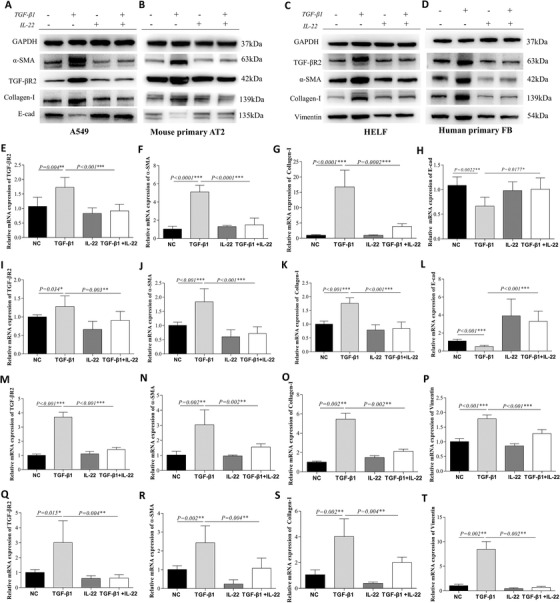
IL‐22 treatment repressed the transforming growth factor (TGF)‐β pathway and the production of collagen in vitro. Samples were collected at 24, 48, and 72 h after treatment cells with 10 ng/ml human interleukin (IL)‐22 and 5 ng/ml TGF‐β1 for mRNA or protein analysis (*n* = 3). (A–B) In both A549 and mouse primary AT2 cells, the expressions of TGF‐βR2, α‐smooth muscle actin (α‐SMA), Collagen‐I, and E‐cad after treatment with TGF‐β1 and/or IL‐22 by WB. (C–D) In both HELF and human primary fibroblasts (FB), the expressions of TGF‐βR2, α‐SMA, Collagen‐I and Vimentin after treatment with TGF‐β1 and/or IL‐22 by WB. (E–L) The mRNA expressions of TGF‐βR2, α‐SMA, Collagen‐I and E‐cad after treatment with TGF‐β1 and/or IL‐22 by real‐time PCR in both type II AECs (A549 cell line and mouse primary AT2). (M–T) The mRNA expressions of TGF‐βR2, α‐SMA, Collagen‐I, and Vimentin after treatment with TGF‐β1 and/or IL‐22 in both fibroblasts (HELF cell line and human primary FB) by real‐time PCR

**FIGURE 6:**
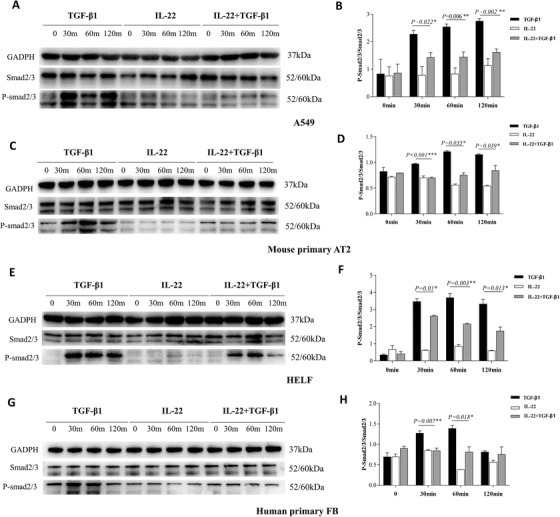
Interleukin (IL)‐22 treatment decreased the activation of Smad2/3 signaling in vitro. The logarithmic growth phase cells were treated with 10 ng/ml rhIL‐22 and 5 ng/ml rhTGF‐β1 and then collected after treatment for 0, 30, 60, and 120 min to analyze the expressions of protein. Each test was repeated three times. (A) The protein expressions of P‐smad2/3 after treatment with TGF‐β1 and/or IL‐22 by WB in A549 cell line. (B) The quantification of (A). (C–D) The results in mouse primary AECs were similar to the A549 cell line. (E) The protein expressions of P‐smad2/3 after treatment with TGF‐β1 and/or IL‐22 by WB in HELF cell line. (F) The quantification of (E). (G–H) The results in primary fibroblasts (FB) were similar to HELF cell line

### 3.4 IL‐22 augmentation ameliorated BLM induced inflammation and collagen deposition

Because the lack of IL‐22 exacerbated pathology in the fibrosis model, we further tested if IL‐22 augmentation could be protective. In this study, we treated the mice with recombinant IL‐22 by intranasal instillation after the BLM challenge (Figure 4A). Indeed, BLM‐induced inflammation (Figure 4B7–9) and collagen deposition (Figure 4C7–9) were significantly ameliorated by IL22 when compared with BLM treated WT mice (Figure 4B4–6, C4–6). The low magnification of lung tissues in both BLM and IL‐22 treated mice is shown in Figure S2–3. The pathological scores of alveolitis at day 7 (*p* = 0.011, Figure 4D) and fibrosis at day 21 (*p* = 0.007, Figure 4E) were significantly decreased in mice treated with both BLM and IL‐22 (*n* = 6) as compared with BLM mice (*n* = 6). IL‐22 augmentation significantly downregulated mRNA expressions of TGF‐β1 and TGF‐βR2 at day 21 (Figure 4F, G) in BLM models. At the molecular level, IL‐22 treatment also reduced the protein expressions of TGF‐β1 and TGF‐βR2 at days 14 and 21 (Figure 4H–J). The protein expressions of P‐smad2/3 (Figure 4K–M), α‐SMA, Collagen‐I, and FN (Figure 4N–P) were also down‐regulated. As shown in Figure S4, the protein expressions of TGF‐β_1_, TGF‐βR2, FN, Collagen‐I, α‐SMA, and P‐SMAD2/3 induced By BLM were also significantly reduced at day 21, even if IL‐22 was administrated at day 7. IL‐22 treatment unexpectedly enhanced Smad activation at the early stage (7 days), despite its potent inhibitory effects at the late time points (14 and 21 days). The underlying mechanism was unclear but likely due to the proinflammatory function of IL‐22^15^ in certain contexts. Nonetheless, IL‐22 markedly inhibited Smad activation at the fibrotic phase of the BLM model. Overall, these data provide evidence that IL‐22 augmentation by intranasal instillation could repress the TGF‐β pathway and BLM‐induced pulmonary fibrosis.

### 3.5 IL‐22 treatment repressed TGF‐β pathway in vitro

To explore the potential mechanism through which IL‐22 protected against fibrosis, we utilized cell culture models. TGF‐β1 was used to treat lung AT2 like cell line (A549), primary mouse AT2 cells, HELF, or primary FB cells. Treatment of TGF‐β1 significantly increased the mRNA (Figure 5F, G) and protein (Figure 5A) expression of α‐SMA and Collagen‐I but decreased E‐Cad (Figure 5A, H) in A549 and also in primary mouse AT2 cells (Figure 5B, J–L), suggesting an epithelial‐mesenchymal‐transition. When IL‐22 was administrated after the TGF‐β1 treatment for 24 h, it also repressed the expression of Collagen‐I and α‐SMA in A549 (Figure S5). In HELF cells, TGF‐β1 increased the mRNA (Figure 5M–P) and protein (Figure 5C) expression of α‐SMA, Collagen‐I, and Vimentin. This finding was consistent with primary FB cells (Figure 5D, R–T). These observations suggest a fibroblast‐myofibroblast‐transition. Both processes have been established to contribute to pulmonary fibrosis^23^, ^24^ ^.^ Interestingly, treatments of IL‐22 partially reversed these processes as demonstrated by its effect on reducing α‐SMA and Collagen‐I expression, partially restoring E‐Cad expression in both A549 and primary AT2 cells (Figure 5A, B, F–H, J–L) and decreasing Vimentin expressing in both FB cells (Figure 5C, D, N–P, R–T). Furthermore, IL‐22 was found to repress TGF‐βR2 mRNA and protein in A549 (Figure 5A, E) and primary AT2 cells (Figure 5B, I), HELF (Figure 5C, M), and primary FB (Figure 5D, Q). The Il22RA1 is expressed in both A549 and HELF cell lines (Figure S6).

Consistently, P‐smad2/3, the key transcriptional factors and signaling molecule downstream of TGF‐β1, was also reduced by IL‐22 treatment in A549 (Figure 6A, B), primary mouse AT2 (Figure 6C, D), HELF (Figure 6E, F), and primary FB cells (Figure 6G, H). Thus, the anti‐fibrotic effect of IL‐22 was likely mediated via direct inhibition of the TGF‐β pathway.

## 4 DISCUSSION

IPF is a progressive fibrotic interstitial lung disease characterized by dysregulated fibroblast proliferation and excessive extracellular matrix deposition, leading to irreversible scarring and loss of pulmonary function. In the present study, we have demonstrated that the expression level of IL‐22 is inversely related to IPF severity (CT score and pulmonary function test). This finding, for the first time, directly links IL‐22 with IPF and suggests that IL‐22 may be protective. To further established cause and effect, we utilized two complementary approaches: IL‐22R^−/−^ and IL‐22 augmentation. In a mouse model of BLM‐induced lung fibrosis, the lack of IL‐22 signaling exacerbated and IL‐22 augmentation prevented inflammation and fibrogenesis, supporting the protective function of IL‐22.

The role of IL‐22 in fibrotic disease models has been controversial. Both pathogenic^15^ and protective^13^, ^14^, ^15^ functions of IL‐22 were reported. The dual role (pathogenic versus protective) of IL‐22 was thought to be governed by the presence and absence of IL‐17A, respectively. The window of this observation was made at an early inflammatory phase (day 10) of the BLM model.^15^ Our study extended this finding by examining both the early inflammatory phase (day 7 post‐BLM challenge) and the later fibrotic phase (day 21). Contrary to their finding, IL‐22‐mediated signaling in our study was found to be consistently protective against inflammation and fibrosis. There are two main differences between our and their studies. First, we used an IL‐22R KO model, but they used the IL‐22 KO mice. Although the interaction between IL‐22 and IL‐22R is very specific, IL‐22R has also been shown to mediate the signaling from IL‐20 or IL‐24.^25^ However, a non‐IL‐22 effect might exist, but likely not an explanation for this discrepancy because the reciprocal experiment using IL‐22 augmentation provided additional support for the protective function of IL‐22 in the entire time course. Second, our mice were derived from a C57/B6 background, but theirs were on a BALB/cBy background. Interestingly, BALB/cBy was found to carry a high‐affinity aryl hydrocarbon receptor (AHR)^26^ that is different from the regular affinity AHR in C57/B6. Because AHR signaling had a high impact on IL‐17/IL‐22 function,^27^ the paradoxical function of IL‐22 in the BLM model may be due to different background levels of AHR signaling. This notion will require further investigation.

Although there was a clear correlation between lower plasma IL‐22 and IPF severity, the cellular source of IL‐22 was not defined. We found a lower level of IL‐22 mRNA in PBMCs from peripheral blood, suggesting that one or more cell types in PBMCs might be the cause of lower IL‐22. IL‐22 was first found to be produced by CD4 T cells^28^ and later associated with Th17 cells.^29^ Th22 has been coined for CD4 helper T cells that produce IL‐22. These cells may also produce IL‐17 and/or IFN‐γ. Other major sources of IL‐22 are the newly found innate lymphoid cells such as ILC3. This rare cell type was found partly because of very high IL‐22 production.^30^ ILC3 also expresses IL‐17.^31^ In our study, lower IL‐17 expressing cells were also observed in IPF patients by flow cytometry (data not shown), suggesting that the potential sources of IL‐22 might be Th17 and/or ILC3. Consistent with our finding, peripheral depletion of Th17 was reported before in IPF patients.^32^ In addition, many other cell types such as γδ T cells^8^ and macrophages^9^ also produced IL‐22. Whether or not these cell types contribute to IL‐22 in IPF is unclear.

Cells recognize IL‐22 by the IL‐22 receptor complex (IL‐22R1 and IL‐10Rβ) that is universally present on both hematopoietic and structural cells. The interaction between IL‐22 and its receptor leads to activation of downstream kinases such as JAK1, TYK2, and MAPKs, thereby further activating transcriptional factors including STAT1, 3, 5, and AP1 family.^33^ In our study, we tested epithelial cells and fibroblasts, two major contributors to IPF pathogenesis. The overall effect of IL‐22 was to inhibit the TGF‐β pathway. TGF‐β signaling initiates from a direct binding between TGF‐β1 and the type 2 receptor (TGF‐βR2), a constitutively active receptor, which leads to the recruitment, phosphorylation, and activation of the type 1 receptor (TGF‐βR1). TGF‐βR1 then phosphorylates downstream transcriptional factors such as Smad2 and 3 at the SS*X*S motif in their C‐tails.^34^ We found that IL‐22 transcriptionally downregulated TGF‐βR2 in vitro and in vivo, which might be responsible for the inhibitory effect of IL‐22 on the TGF‐β pathway. This type of TGF‐βR2 downregulation was reported before in human squamous cell carcinoma,^35^ cervical cancer^36^, or in female T effector cells.^37^ Although the underlying mechanism is not clear at the moment, MAPK activation, a downstream event of IL‐22, was previously shown to repress TGF‐βR2 expression in intestinal epithelial cells.^38^ However, the precise role of MAPK activation is unclear, as it was reported to modulate TGF‐β signaling agonistically or antagonistically under different conditions,^39^ Additionally, KLF14, a downstream transcription factor of TGF‐βR2, was shown to induce transcriptional silencing of TGF‐βR2 via feedback control.^40^ Thus, KLF14 might be enhanced by IL‐22 for suppressing TGF‐βR2 transcription.^40^ Nonetheless, further research will be needed to test these possibilities.

In our study, IL‐22 augmentation could ameliorate BLM‐induced inflammation and fibrosis. Thus, IL‐22 enhancement may be a viable approach to combat fibrotic diseases such as IPF. To date, most effects on the drug development targeting IL‐22 have been focused on its inhibition by neutralizing antibodies. A recent Phase 2 clinical trial showed the efficacy of an IL‐22‐neutralizing antibody (fezakinumab) in atopic dermatitis.^41^ To enhance IL‐22, recombinant IL‐22 with different modifications aiming to increase its in vivo stability is in the process of development.^42^, ^43^, ^44^ A human IL‐22Fc fusion protein (UTTR1147A) developed by Genetech completed a phase I trial with acceptable safety, pharmacokinetics, and IL‐22 engagement in healthy volunteers.^42^ Interestingly, a different approach using probiotic *Lactobacillus* to deliver bioactive IL‐22 was proposed and tested to benefit graft‐versus‐host disease patients.^45^ Therefore, both IL‐22Fc fusion protein and IL‐22 bearing *Lactobacillus* can be further developed to enhance the pulmonary expression of IL‐22 for the treatment of IPF.

In summary, we have demonstrated for the first time that IL‐22 expression was inversely associated with IPF severity in a clinical cohort. The cause and effect of IL‐22 in the protection against pulmonary fibrosis have been established by using an IL‐22R KO mouse model and an IL‐22 protein augmentation model via intranasal instillation of recombinant IL‐22. Both in vitro and in vivo studies have further demonstrated that the protective function of IL‐22 signaling may be due to its inhibitory effect on the TGF‐β pathway. Therefore, IL‐22 is protective against fibrosis, and IL‐22 enhancement may be a novel approach to treat IPF.

## CONFLICT OF INTEREST

All authors have declared no conflict of interest.

## AUTHOR CONTRIBUTIONS

Mengshu Cao, Dandan Wang, and Yin Chen conceived and prepared the manuscript. Mengshu Cao, Dandan Wang, Peiyu Gu, Xin Wang, Zhiyong Chen, and Lina Gu contributed to the collection of clinical data, statistics, and experiments. Mengshu Cao, Lina Gu, Mengying Liu, Fanqing Meng, and Jun Yang evaluated and scored the histopathology. Fanqing Meng and Ji Zhang provided the lung tissue sample. Mengshu Cao, Peiyu Gu, Xin Wang, Lina Gu, Yonglong Xiao, and Hourong Cai contributed to administrate the patients. Mengshu Cao and Yin Chen designed the study, reviewed the manuscript, had full access to all of the data, and took responsibility for the integrity of the data and the accuracy of the data analysis. All authors read and approved the final draft.

## Supporting information



Supporting Information

Supporting Information

Supporting Information
