# Supplementary material for: Protective function of interleukin‐22 in pulmonary fibrosis
Source: Clin Transl Med. 2021 Aug 26;11(8):e509. doi: 10.1002/ctm2.509 (PMC8387792; doi:10.1002/ctm2.509)

Supplemental Figure. 1

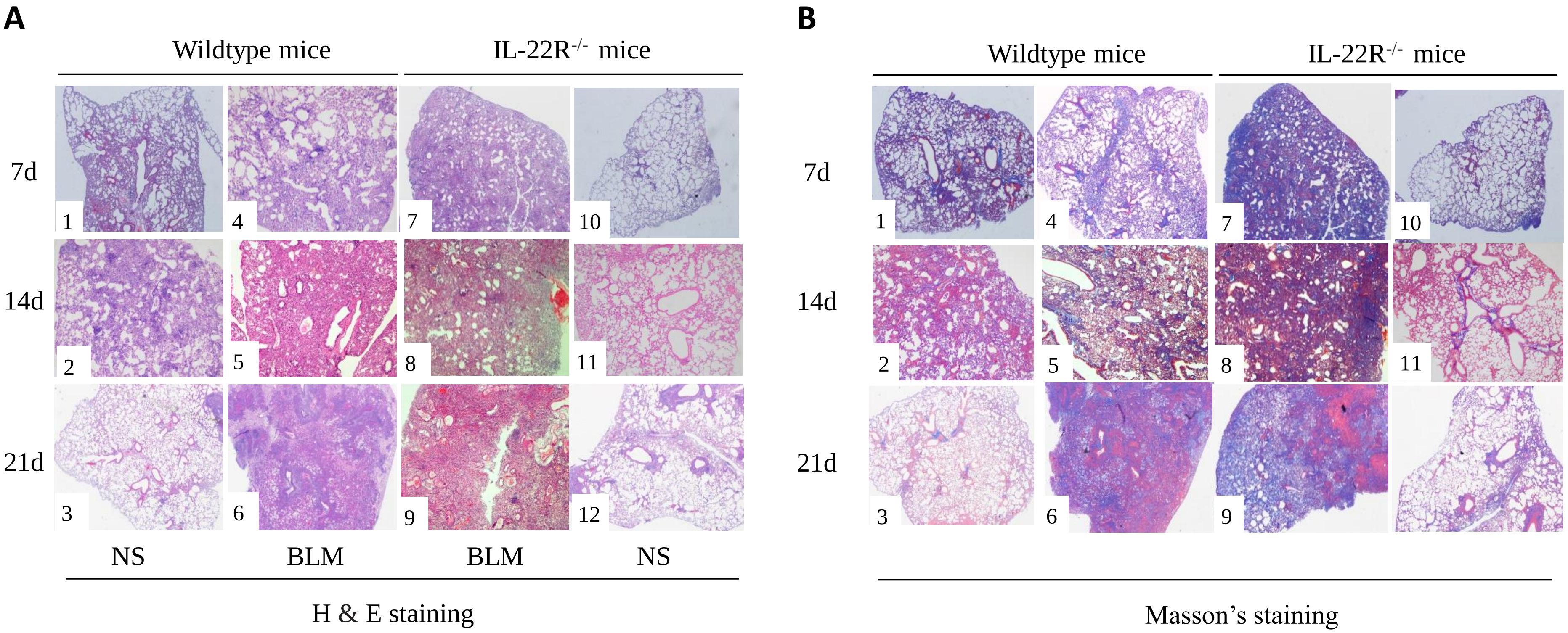

Supplemental Figure. 2

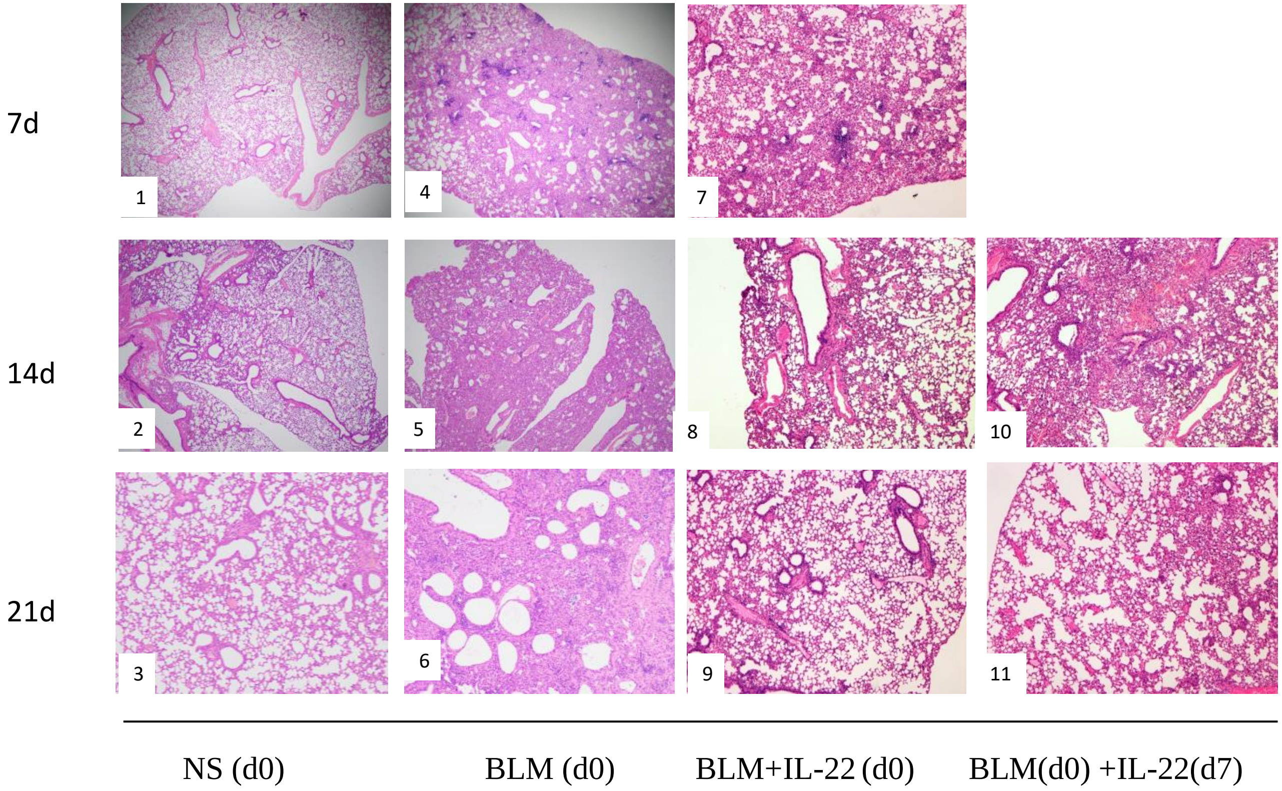

HE staining

Supplemental Figure. 3

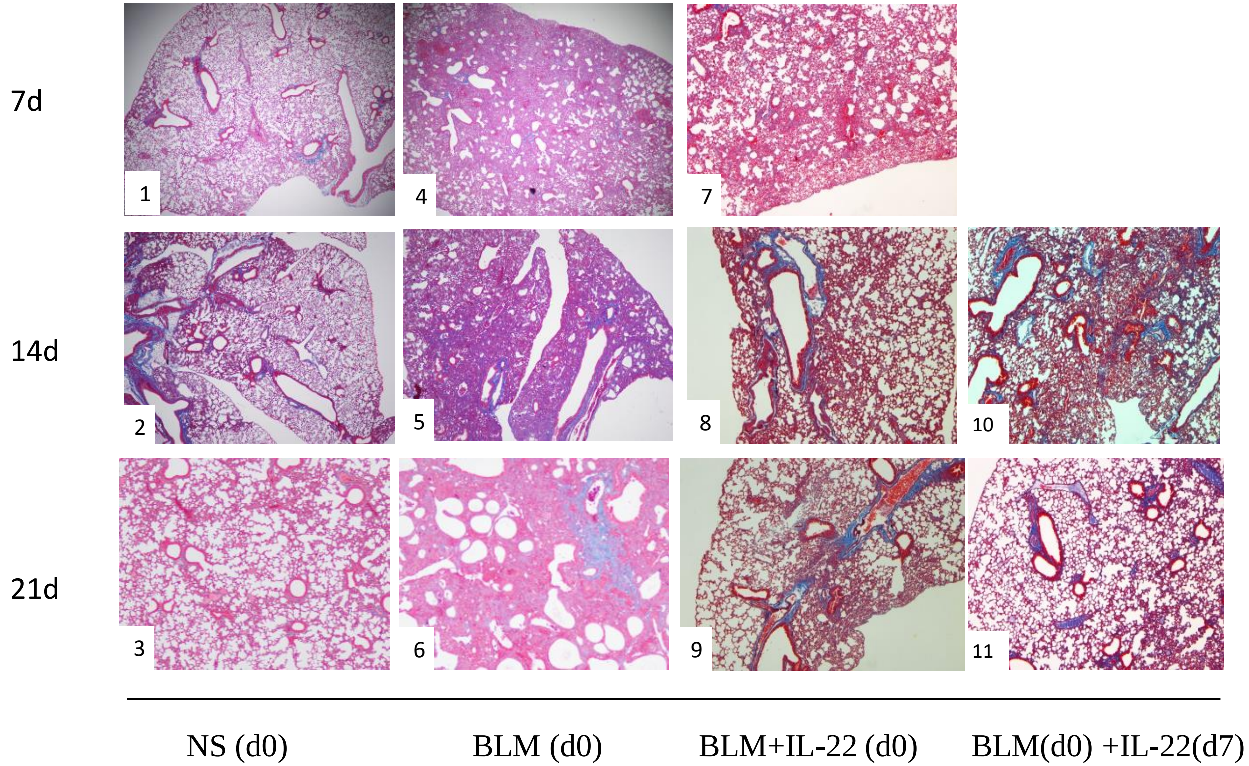

Masson's staining

Supplemental Figure. 4

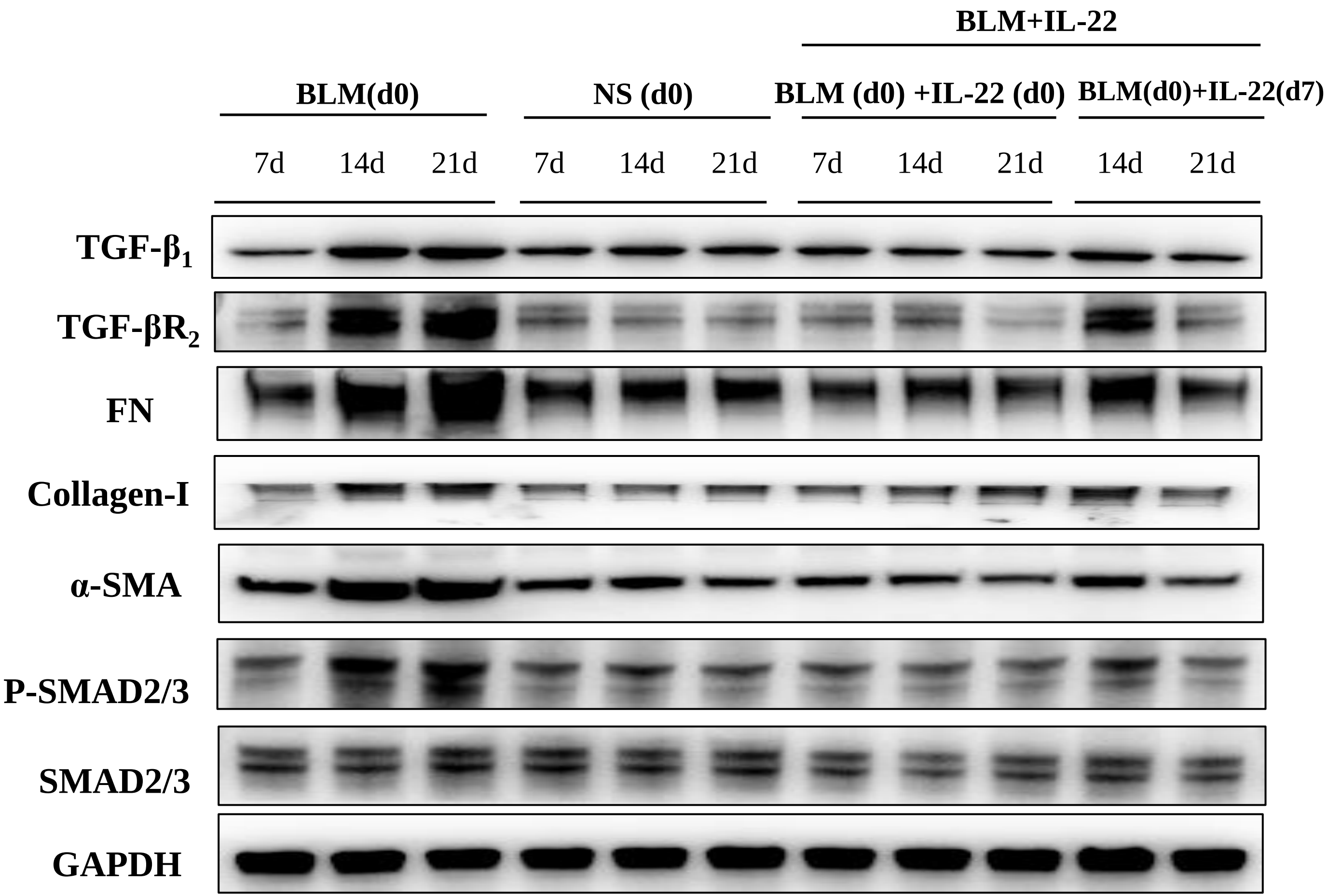

Supplemental Figure. 5

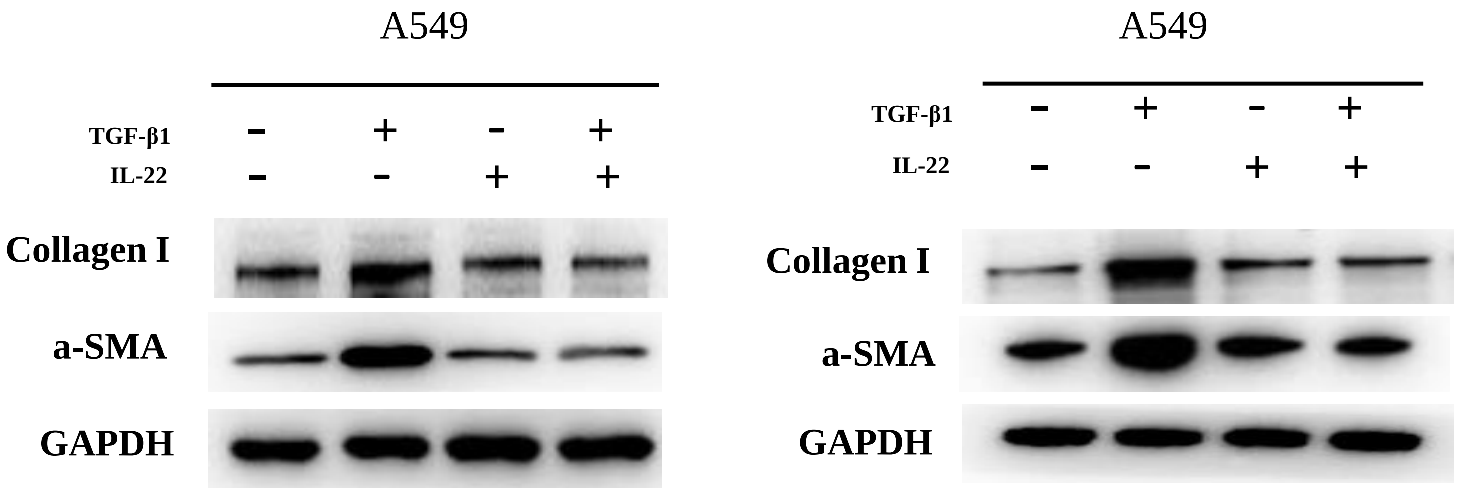

Supplemental Figure. 6

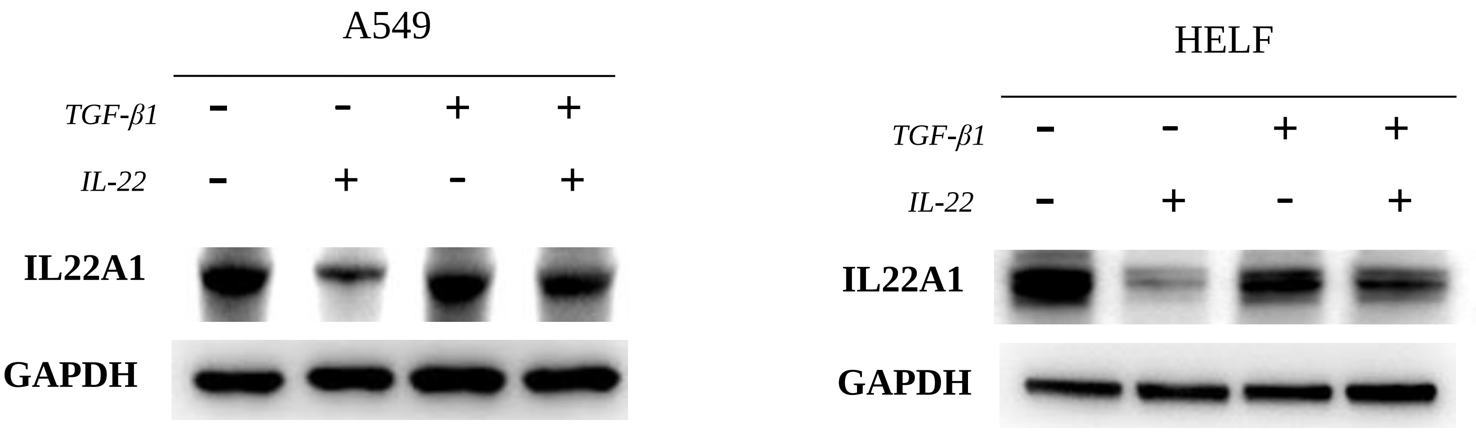

Supplement: Supplementary file 1 — Supporting Information [file CTM2-11-e509-s003.pdf]
