# Supplementary material for: Protective function of interleukin‐22 in pulmonary fibrosis
Source: Clin Transl Med. 2021 Aug 26;11(8):e509. doi: 10.1002/ctm2.509 (PMC8387792; doi:10.1002/ctm2.509)
Supplement: Supplementary file 2 — Supporting Information [file CTM2-11-e509-s001.docx]

**Supplemental Table 1. Baseline clinical characteristics of IPF patients*.***

|  | **IPF patients** |
| --- | --- |
| Gender (M/F) | 21/3 |
| Age (years old) | 67.2±9.8 |
| IPF duration (m) | 32 (1-80) |
| Smoking(Y/N) | 13/11（54.17%） |
| Smoking amount （pack-years） | 26.35（2.5-50） |
| Comorbidities  respiratory failure  diabetes  hypertension  Coronary heart disease  liver cirrhosis  malignancy | 11/24 (45.83%)  6/24 (25%)  5/24 (20.83%)  2/24 (8.33%)  2/24 (8.33%)  2/24 (8.33%) |
| FVC (L) | 2.12±0.82 |
| FVC % pred | 61.48±13.52 |
| FEV1 % pred | 72.44±14.17 |
| TLC % pred | 49.13±10.70 |
| DLCO % pred | 42.27±18.68 |
| CT scores | 5.15±1.98 |
