# Supplementary material for: Protective function of interleukin‐22 in pulmonary fibrosis
Source: Clin Transl Med. 2021 Aug 26;11(8):e509. doi: 10.1002/ctm2.509 (PMC8387792; doi:10.1002/ctm2.509)
Supplement: Supplementary file 3 — Supporting Information [file CTM2-11-e509-s002.docx]

**Supplemental Figure 1. Low-magnification pictures of lung tissues of wild type and IL-22R ^-/-^mice as described in Figure. 3.**

A1-6: The pathological changes of lung tissues at day 7, 14 and 21 days in NS and BLM treated WT mice. A7-12: The pathological changes of lung tissues at day 7, 14 and 21 days in BLM and NS treated IL-22R**^-/-^** mice. A1-12: HE staining (×40). B1-6: The pathological changes of lung tissues at day 7, 14 and 21 days in NS and BLM treated WT mice. B7-12: The pathological changes of lung tissues at day 7, 14 and 21 days in BLM and NS treated IL-22R**^-/-^** mice. B1-12: MS staining (×40). NS: normal saline. BLM: bleomycin.

**Supplemental Figure 2. Low-magnification pictures of lung tissues in BLM and/or IL-22 treated mice as described in Figure 4 by HE staining.**

A1-3: The pathological changes of lung tissues at day 7, 14 and 21 days in NS (d0) treated mice. A4-6: The pathological changes of lung tissues at day 7, 14 and 21 days in BLM (d0) treated mice. A7-9: The pathological changes of lung tissues at day 7, 14 and 21 days in both BLM (d0) and IL-22 (d0) treated mice at the same time. A10-11: The pathological changes of lung tissues at days 14 and 21 days in both BLM (d0) and IL-22 (d7) treated mice at the different time. A1-11: HE staining (×40).

**Supplemental Figure 3. Low-magnification pictures of lung tissues in BLM and/or IL-22 treated mice as described in Figure 4 by MS staining.**

B1-3: The pathological changes of lung tissues at day 7, 14 and 21 days in NS (d0) treated mice. B4-6: The pathological changes of lung tissues at day 7, 14 and 21 days in BLM (d0) treated mice. B7-9: The pathological changes of lung tissues at day 7, 14 and 21 days in both BLM (d0) and IL-22 (d0) treated mice at the same time. B10-11: The pathological changes of lung tissues at days 14 and 21 days in both BLM (d0) and IL-22 (d7) treated mice at the different time. B1-11: MS staining (×40).

**Supplemental Figure 4. IL-22 ameliorated BLM induced expression of fibrotic markers and production of Collagen-I and repressed expression of P-smad2/3 in mice.**

The findings of WB showed that the protein expressions of TGF-β_1_, TGF-βR2, FN, Collagen-I, α-SMA and P-SMAD2/3 induced by BLM were also significantly reduced at day 21, even if IL-22 was administrated at day 7.

BLM+IL-22: co-treatment of BLM and IL-22. BLM (d0) + IL-22(d0): Mice were treated with BLM and IL-22 at day 0. BLM (d0) + IL-22(d7): Mice were treated with BLM at day 0 and IL-22 at day 7.

**Supplemental Figure 5. IL-22 repressed the expression of Collagen-I and α-SMA in A549.**

A: IL-22 and TGF-β1 were used to treat A549 cells at the same time. B: IL-22 was used to treat A549 cells after TGF-β1 treatment for 24 hours.

**Supplemental Figure 6. The expression of IL-22RA1 in A549 and HELF cell lines by WB.**

A: The expression of IL-22RA1 after treatment with TGF-β1 and/or IL-22 in A549 cell line. B: The expression of IL-22RA1 after treatment with TGF-β1 and/or IL-22 in HELF cell line.
